# α‐Synuclein decoy peptide protects mice against α‐synuclein‐induced memory loss

**DOI:** 10.1111/cns.14120

**Published:** 2023-02-14

**Authors:** Qingyun Guo, Ichiro Kawahata, Wenbin Jia, Haoyang Wang, An Cheng, Yasushi Yabuki, Norifumi Shioda, Kohji Fukunaga

**Affiliations:** ^1^ Key Laboratory of Brain Science Research & Transformation in Tropical Environment of Hainan Province Hainan Medical University Haikou China; ^2^ Department of CNS Drug Innovation, Graduate School of Pharmaceutical Sciences Tohoku University Sendai Japan; ^3^ Department of Genomic Neurology Institute of Molecular Embryology and Genetics, Kumamoto University Kumamoto Japan; ^4^ BRI Pharma Incorporated Sendai Japan

**Keywords:** decoy peptide, fatty acid‐binding proteins 3, memory impairment, αSyn‐FABP3 complex, α‐synuclein

## Abstract

**Aims:**

We previously found that a decoy peptide derived from the C‐terminal sequence of α‐Synuclein (αSyn) prevents cytotoxic αSyn aggregation caused by fatty acid‐binding protein 3 (FABP3) in vitro. In this study, we continued to utilize αSyn‐derived peptides to further validate their effects on αSyn neurotoxicity and behavioral impairments in αSyn preformed fibrils (PFFs)‐injected mouse model of Parkinson's disease (PD).

**Methods:**

Mice were injected with αSyn PFFs in the bilateral olfactory bulb (OB) and then were subjected to behavioral analysis at 2‐week intervals post‐injection. Peptides nasal administration was initiated one week after injection. Changes in phosphorylation of αSyn and neuronal damage in the OB were measured using immunostaining at week 4. The effect of peptides on the interaction between αSyn and FABP3 was examined using co‐immunoprecipitation.

**Results:**

αSyn PFF‐injected mice showed significant memory loss but no motor function impairment. Long‐term nasal treatment with peptides effectively prevented memory impairment. In peptide‐treated αSyn PFF‐injected mice, the peptides entered the OB smoothly through the nasal cavity and were mainly concentrated in neurons in the mitral cell layer, significantly suppressing the excessive phosphorylation of αSyn and reducing the formation of αSyn‐FABP3 oligomers, thereby preventing neuronal death. The addition of peptides also blocked the interaction of αSyn and FABP3 at the recombinant protein level, and its effect was strongest at molar concentrations comparable to those of αSyn and FABP3.

**Conclusions:**

Our findings suggest that the αSyn decoy peptide represents a novel therapeutic approach for reducing the accumulation of toxic αSyn‐FABP3 oligomers in the brain, thereby preventing the progression of synucleinopathies.

## Graphical abstract

Decoy peptides derived from the C‐terminal tail of αSyn rescue memory loss caused by αSyn preformed fibrils injection into the mouse olfactory bulb and inhibit αSyn phosphorylation and formation of toxic oligomers.

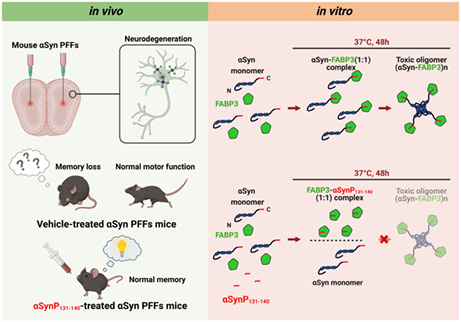

## 1 INTRODUCTION

Synucleinopathies are a group of neurodegenerative disorders, including Parkinson's disease (PD), dementia with Lewy bodies, and multiple system atrophy, characterized by the pathologic aggregation of α‐Synuclein (αSyn) in neurons and glial cells.^1^, ^2^ αSyn is an intrinsically unfolded polypeptide consisting of 140 amino acid residues with three characteristic domains: an amphiphilic charged N‐terminal region (residues 1–60), a central, hydrophobic, non‐amyloid‐β component (NAC) region (residues 61–95), and a C‐terminal acidic domain (residues 96–140).^3^, ^4^, ^5^ The N‐terminal region contains conserved imperfect repeats, crucial for generating a helical structure for membrane interactions.^5^ All previously identified mutations are in this domain and alter the membrane‐bound properties of αSyn without generating major structural changes in the monomer.^6^ In addition, the NAC region is highly amyloidogenic and is thought to form the core of αSyn amyloid fibrils,^7^ whereas the C‐terminal region is rich in acidic residues and prevents αSyn aggregation through electrostatic repulsion.^4^ Therefore, further study of the C‐terminal region is useful for understanding the key steps of the initiation of αSyn oligomerization.

αSyn toxicity is triggered by αSyn oligomerization, and previous studies have demonstrated that αSyn binding to polyunsaturated fatty acids (PUFAs) such as arachidonic acid and docosahexaenoic acid is a critical event in the conversion of soluble monomers to pathological oligomers.^8^, ^9^, ^10^, ^11^ We expanded on these findings and further demonstrated that fatty acid‐binding protein 3 (FABP3) acts as a cellular shuttling of PUFAs, exacerbating arachidonic acid‐induced αSyn oligomerization in dopaminergic neurons.^12^, ^13^ Moreover, FABP3 inhibitors have been shown to reduce αSyn oligomerization in a mouse model of PD induced by injection of 1‐methyl‐4‐phenyl‐1,2,3,6‐tetrahydropyridine^14^, ^15^ or αSyn preformed fibrils (PFFs).^16^, ^17^ FABP3 co‐localization with αSyn aggregates has also been observed in the brains of patients with synucleinopathies.^18^ However, as research has progressed, FABP3 has been found to bind to αSyn and induce it to oligomerize without relying on PUFAs. This interaction involves the C‐terminus of αSyn.^6^ In in vitro αSyn fibrillation assays, the presence of FABP3 contributes to a strong inhibition of αSyn fibril formation by forming a 1:1 binary complex of αSyn and FABP3, leaving αSyn in a state of soluble aggregates [(αSyn‐FABP3)n oligomers], preventing its aggregation into a higher molecular fibril transformation.^19^ This phenomenon is attributed to the blockade of the C‐terminal region of αSyn by FABP3, which enhances the hydrophobicity of the αSyn surface, whereas the conversion of soluble oligomers to fibrils reduces hydrophobicity. Furthermore, we did not detect any significant intracellular deposits of insoluble αSyn in FABP3‐deficient animals or cell models of PD,^16^, ^20^ implying that the initiation of αSyn oligomerization is largely dependent on FABP3. Based on these findings, we hypothesized that a “fake” αSyn (decoy peptide) from the C‐terminal could confuse FABP3 and compete for binding to FABP3, hinder its interaction with “true” αSyn, and avoid the occurrence of αSyn aggregation and toxicity.

According to the Braak and double‐hit hypothesis, sporadic PD may start in the olfactory bulb (OB) and enteric plexus.^21^, ^22^ The widespread transneuronal transmission of αSyn initiated from mouse OB is regarded as a novel prodromal PD model.^23^ Therefore, exogenous αSyn PFFs injection into the mouse OB has been frequently employed to investigate the spreading, targeting, and neurotoxicity of αSyn pathology.^24^, ^25^ In this study, we employed αSyn PFFs‐injected mouse model to examine the neuroprotective potential of αSyn decoy peptides. αSyn neurotoxicity in the OB significantly impaired mouse memory function but did not affect motor performance, and peptide therapy effectively attenuated neuronal damage in the OB to avoid memory impairment.

## 2 MATERIALS AND METHODS

### 2.1 Animals

Male C57BL/6J mice (10‐weeks old) were purchased from Japan SLC, Inc. (Shizuoka, Japan). The animals were housed under the conditions of constant temperature (23 ± 2°C) and humidity (55 ± 5%), with a 12/12‐h light–dark cycle (lights on: 09:00–21:00), and unlimited access to food and water. Mice housed within the same cage (225 × 340 × 155 mm) are considered as one group. There are no environmental enrichment materials or objects in the cages other than bedding. All procedures for handling animals complied with the Guide for Care and Use of Laboratory Animals and were approved by the Animal Experimentation Committee of Tohoku University Graduate School of Pharmaceutical Sciences [2019PhLM0‐021 (approved date: 1 December 2019) and 2019PhA‐024 (approved date: 1 April 2019)].

### 2.2 Intracerebral stereotactic injection of αSyn PFFs

**FIGURE 1:**
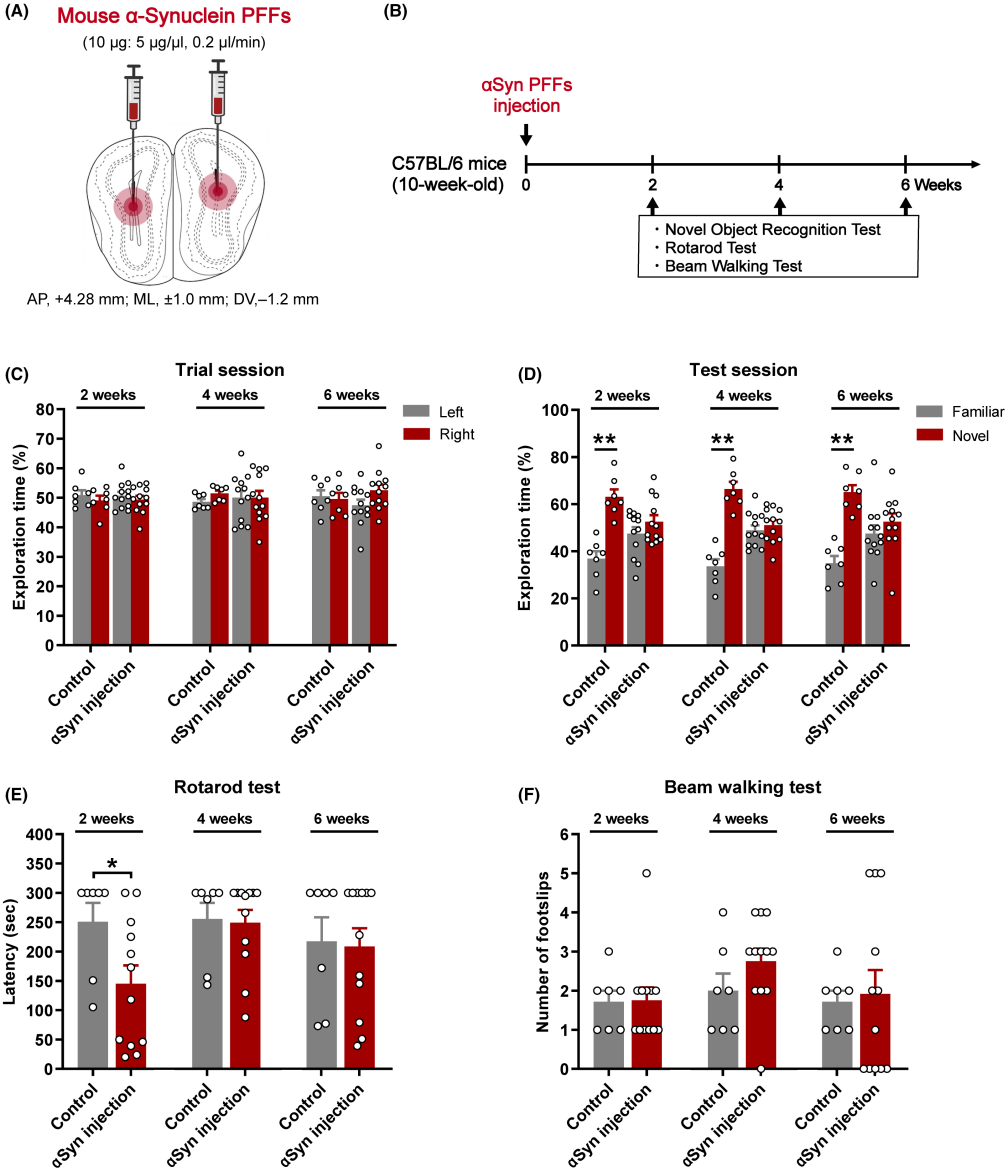
Effects of αSyn PFF injection on memory and motor function in mice. (A) αSyn PFFs were injected into the bilateral olfactory bulb. (B) Experimental schedule for behavioral tests. Mice were subjected to behavioral tests every 2 weeks after injection. (C, D) The percentage of time exploring object during the trial session (C) and the test session (D) in a novel object recognition test at 2, 4, and 6 weeks. The data shown in each column represent the mean ± SEM. **p < 0.01 with a paired Student's *t*‐test (familiar vs. novel for each group). (E, F) Analyses of motor coordination based on a rotarod test and beam walking test at 2, 4, and 6 weeks after injection. The latency before falling from a rolling drum (E) and the number of foot slips before reaching the goal box (F) were recorded. The data shown in each column represent the mean ± SEM. *p < 0.05 vs. control group. (control group: *n* = 7; αSyn injection group: *n* = 12).

Mouse ATTO‐550‐labeled αSyn fibrils were prepared as previously described^20^ and stored at −80°C until use. Injections for αSyn PFFs were performed under general anesthesia with three mixed anesthetics (0.3 mg/kg medetomidine, 4.0 mg/kg midazolam, and 5.0 mg/kg butorphanol).^26^ The mice were fixed on the stereotactic instrument, and then, αSyn PFFs (10 μg; 5 μg/μL) were injected into the bilateral OB (anterior–posterior (AP), +4.28 mm; medial–lateral (ML), ±1.0 mm; dorso–ventral (DV), −1.2 mm) at a slow rate (2 μL per hemisphere at a speed of 0.2 μL/min) using a 10‐μL Hamilton syringe (26 s gauge, 2 inch needle, Hamilton Company, Cat#80300) (Figure 1A). After 10 min of infusion, the needle was held in place for an additional 2 min, then slowly withdrawn, and the skin was sutured. Then, the mice were returned to their cages for recovery. To ensure viral expression, we employed a waiting period of 2 weeks after injection before performing behavioral tests.

### 2.3 Peptide preparation

**FIGURE 2:**
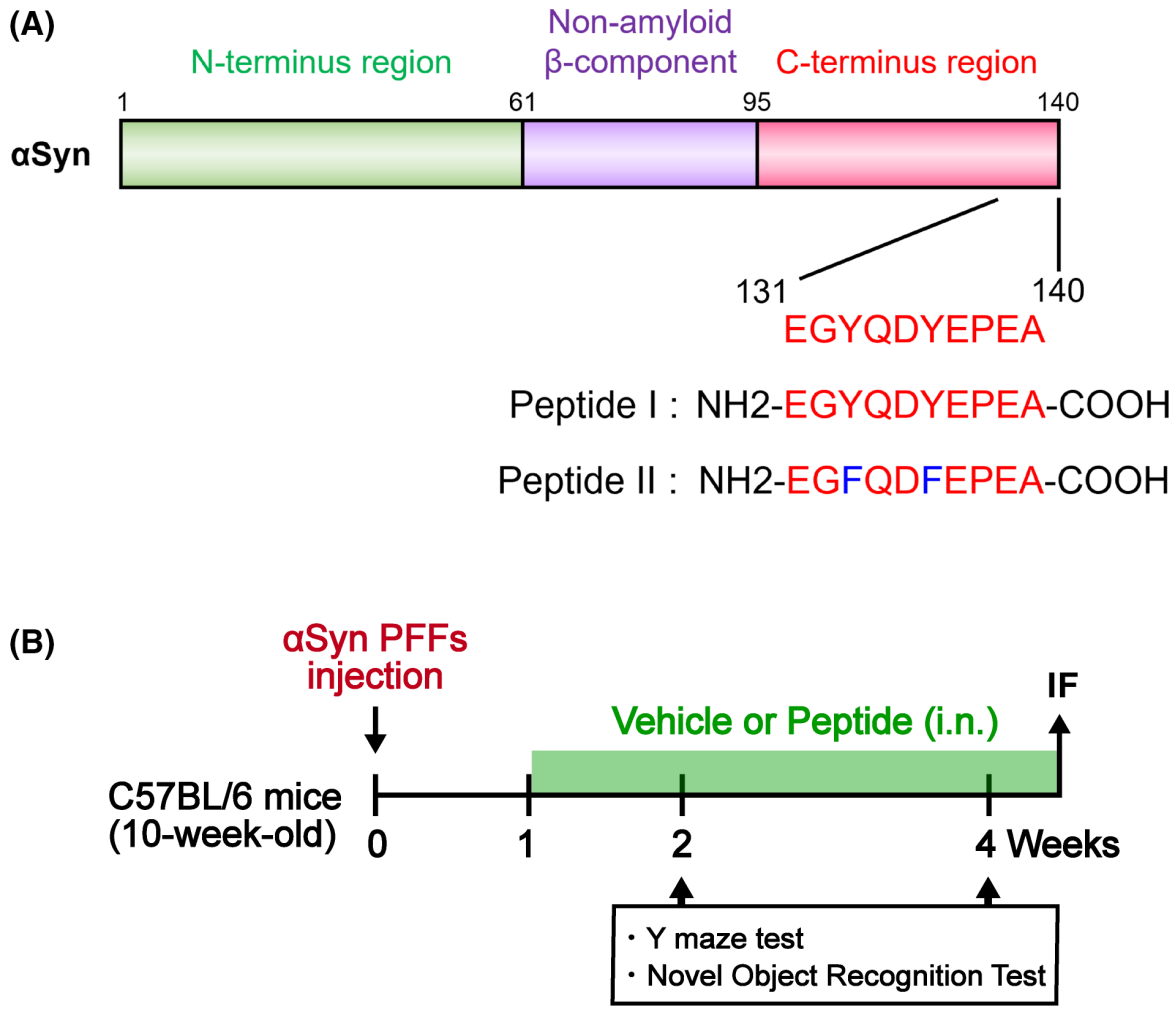
Experimental schedule for peptide treatments and behavioral tests. (A) Chemical synthesis of decoy Peptides I and II according to the amino acid sequence of αSyn131–140. (B) Experimental schedule for peptide treatments and behavioral tests. Nasal peptide treatment was administered 1 week after αSyn PFF injection.

Peptides derived from the αSyn amino acid sequence (αSynP130‐140; EEGYQDYEPEA) were synthesized by Eurofins Genomics (Tokyo, Japan). The three peptides, I (NH2‐EGYQDYEPEA‐COOH; 1200.16 Da; 99.03% purity), II (NH2‐EGFQDFEPEA‐COOH; 1168.17 Da; 95.17% purity), were dissolved in phosphate‐buffered saline (PBS) for ease of use. As shown in Figure 2B, one week after αSyn PFFs injection, mice were administered with peptide (50 or 150 μg/kg) intranasally (i.n.) once daily, or the equivalent volume of PBS (for controls), until sacrifice.

### 2.4 Behavioral tests

#### 2.4.1 Rotarod test

The rotarod test was used to assess motor coordination and balance, as previously described.^27^ The rotarod apparatus consisted of a base platform and a rotating rod with a non‐skid surface (diameter, 3 cm; length, 30 cm). Before PFF injection, the mice were placed on a rod rotating at 20 rpm, and the process was repeated until the fall latency exceeded 100 s. The fall delay time was measured for 300 s during the test session.

#### 2.4.2 Beam walking test

The beam walking test was used to assess whole‐body balance and was performed in accordance with a previously described procedure.^17^ The apparatus was made up of a rectangular beam (length, 870 mm; width, 5 mm) and a goal box (155 mm × 160 mm × 5 mm). Before PFF injection, the mice were habituated to the goal box for 3 min, and then, they were placed on the beam 10 cm from the goal box. After traversing 10 cm and reaching the box, the mice were placed at additional distances of 30, 50, and 80 cm from the box. Mice were judged to meet the criteria if they could move through the entire 80 cm to reach the box within 60 s. During the testing phase, the number of foot slips (missteps) that occurred from the end of the beam before reaching to the goal box was recorded. However, we did not measure the latency of the mouse to traverse the beam because we do not think it is a recognized behavioral paradigm.

#### 2.4.3 Y‐maze test

Short‐term spatial memory was studied using the Y‐maze test, as previously described.^28^ Briefly, a mouse was placed at the end of the Y‐shaped maze arm and allowed to explore the maze freely for 8 min. After each session, the apparatus was cleaned with 70% ethanol to prevent odor identification. Alternation behavior was defined as entries into all three arms in consecutive choices. The percentage of alternations was calculated as actual alternation/(the number of total arm entries − 2) × 100. The total number of arm entries (*N*) was recorded as an index of locomotor activity.

#### 2.4.4 Novel object recognition test

The novel object recognition test for evaluating cognitive function was performed, as previously described.^29^ In the trial session, the mice were exposed to two objects of the same shape and size, placed symmetrically in the center of the open field box (35 × 25 × 35 cm^3^) for 10 min. At 1‐h intervals, one object was replaced with a novel object, and exploratory behavior was monitored for 5 min (test session). Exploration of an object was defined as holding up, touching, or sniffing at a distance of <1 cm from the object. The discrimination ratio was calculated as the time spent exploring the novel object or the familiar object divided by the total time spent with both novel and familiar objects.

### 2.5 αSyn‐FABP3 binding assay

The protocols used to obtain purified αSyn protein and FABP3 protein were previously described.^19^, ^30^ Different concentrations of the peptide were pre‐incubated with FABP3 protein (30 μM) at 37°C for 2–6 h, and then, αSyn protein (30 μM) was added to continue incubation for 48 h. After incubation, an anti‐FABP3 antibody (Proteintech, Cat# 10676‐1‐AP, RRID: AB_2102309) was added for 4 h to capture the αSyn‐FABP3 complex, followed by immunoprecipitation with protein A‐Sepharose overnight at 4°C with gentle shaking. Samples were then washed three times with PBS and separated using sodium dodecyl sulfate‐polyacrylamide gel electrophoresis using commercially available gels (Cosmo Bio Co., Ltd.). After electrophoresis, the gels were transferred to polyvinylidene difluoride membrane. Following blocking with T‐TBS solution containing 5% fat‐free milk for 1 h at room temperature, the membranes were incubated with mouse anti‐alpha‐Synuclein antibody, clone 4B12 (1:1000, GeneTex, Cat# GTX21904, RRID: AB 380314) overnight at 4°C with gentle shaking. After washing, the membranes were incubated with secondary antibody for 2 h at room temperature. The membranes were developed using an enhanced chemiluminescence (ECL) immunoblotting detection system (Amersham Biosciences) and visualized using a Luminescent Image Analyzer (LAS‐4000 mini; Fuji Film). The densities of the protein bands were quantified using ImageJ Gauge software (version 3.41; Fuji Film).

### 2.6 Immunofluorescence staining

The immunofluorescence staining was performed as previously described.^26^ The mice were anesthetized and transcardially perfused with ice‐cold PBS, immediately followed by 4% PFA. Mouse brains were extracted and fixed in 4% PFA overnight at 4°C. OB tissues were sliced into 50‐μm coronal sections using a vibratome (Dosaka EM Co. Ltd.). Then, the sections were washed in PBS for 30 min, permeabilized in PBS containing 0.1% Triton X‐100 for 2 h, and blocked in PBS containing 1% bovine serum albumin and 0.3% Triton X‐100 for 1 h at room temperature. The sections were then incubated for 3 days at 4°C with the following primary antibodies: rabbit anti‐FABP3 antibody (1:200, Proteintech, Cat# 10676‐1‐AP, RRID: AB_2102309), mouse anti‐α‐Synuclein Phospho (Ser129) antibody (1:20,000, Biolegend, Cat# 825701, RRID: AB_2564891), rabbit anti‐MAP2 antibody (1:500, Cell Signaling Technology, Cat# 4542 S, RRID: AB_10693782), and mouse anti‐αSyn C‐terminus antibody (1:500, homemade). After washing with PBS, the sections were incubated with Alexa Flour‐conjugated secondary antibodies overnight at 4°C. After extensive washing with PBS, the brain sections were mounted on slides with Vectashield (Vector Laboratories, Inc.). Immunofluorescent images were analyzed using a confocal laser scanning microscope (Nikon).

### 2.7 Statistical analysis

Animal group sizes were chosen based on a priori power analysis (*n* = 5–12 per group, G*Power software version 3.1.9.7, *F*‐tests: medium effect = 0.25, large effect = 0.40, power = 0.90, and α = 0.05) and extensive previous experience with the animal models used. Post hoc power analysis confirmed that our sample size in behavioral test provided sufficient power to detect this effect (power > 97%; ANOVA: Repeated measures, between factors). Any animal that died or was severely injured during the experiment was excluded from study, but this did not occur in this study. No data points were excluded from the analysis in any experiment.

Data analysis was conducted using GraphPad Prism 8 (GraphPad Software, Inc.). All data were first tested for normality using the Shapiro–Wilk normality test. Normally distributed data: Student's *t*‐test was used to analyze differences between two groups (control group and vehicle‐treated αSyn PFFs group), and differences between multiple groups (vehicle‐ or peptides‐treated αSyn PFFs groups) were tested using one‐way analysis of variance (ANOVA) with Dunnett's post hoc method, in case of normally distributed data. Non‐parametric testing, Mann–Whitney Rank Sum test for two groups or the Kruskal‐Wallis test followed by Dunn's multiple comparisons test were performed on data that did not pass normality testing. Paired data analysis was performed using the paired *t*‐test. Data were shown as the mean ± standard error of the mean (SEM). A value of *p* < 0.05 was considered to reflect statistically significant differences.

## 3 RESULTS

### 3.1 αSyn PFFs injected into OB caused memory loss without motor impairment in mice

We examined whether αSyn aggregation in the OB affects PD‐related behavior, including memory and motor function, in mice. Behavioral changes in mice were evaluated every 2 weeks after PFF injection (Figure 1B). In the novel object recognition test, no difference was observed in the percentage of time to discriminate the same object among all groups during the trial session (Figure 1C). However, in the test session, the control mice spent more time on the novel object, while αSyn PFF‐injected mice failed to distinguish between familiar and novel objects from 2 weeks until the last observation (2 weeks: *p* = 0.385, *t* = −0.904, df = 11; 4 weeks: *p* = 0.614, *t* = −0.520, df = 11; 6 weeks: *p* = 0.492, *t* = −0.710, df = 11; Figure 1D). In contrast, αSyn PFF injection into the OB did not result in stable motor dysfunction in a short period of time. In the rotarod test, the latency time of stopping the rotarod in αSyn PFF‐injected mice did not differ significantly from that of the control mice, except at 2 weeks (2 weeks: *p* = 0.0417; 4 weeks: *p* = 0.863; 6 weeks: *p* = 0.863; vs. control; Figure 1E). Furthermore, αSyn PFF injection did not significantly increase the frequency of foot slips in the beam walking test (2 weeks: *p* = 0.942; 4 weeks: *p* = 0.186; 6 weeks: *p* = 0.811; vs. control; Figure 1F). Together, these results revealed that αSyn propagation in the OB causes memory loss in mice, but not motor dysfunction.

### 3.2 αSyn peptides improved the memory impairment of αSyn PFF‐injected mice

**FIGURE 3:**
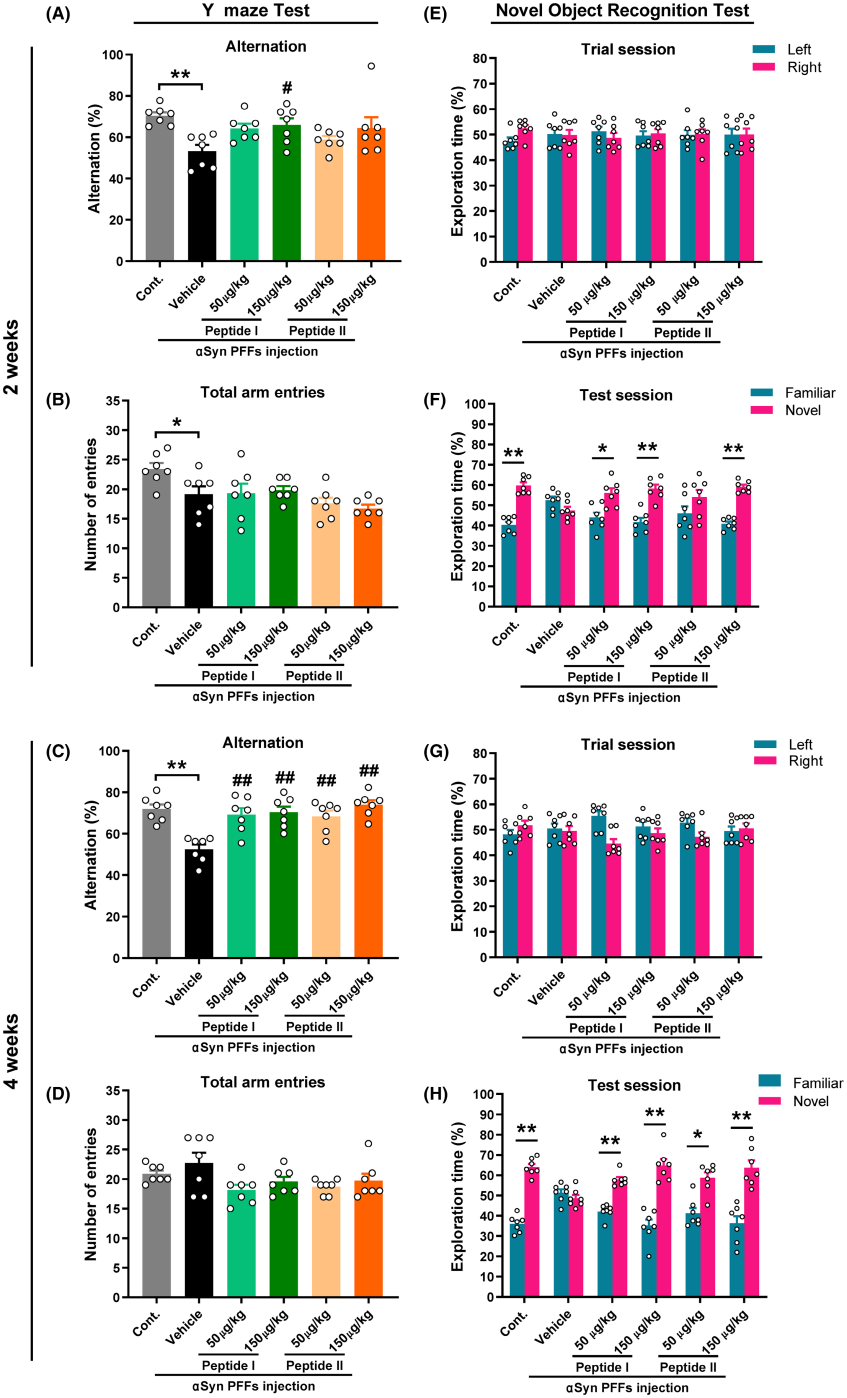
Effects of peptide treatment on αSyn PFF‐induced memory deficits in mice. (A–D) Alternations (A, C) and number of total arm entries (B, D) in a Y‐maze task at 2 and 4 weeks (*n* = 7 per group). The data shown in each column represent the mean ± SEM. *p < 0.05, **p < 0.01 vs. control group; #*p* < 0.05, ##*p* < 0.01 vs. vehicle‐treated αSyn PFF injection group. (E–H) The percentage of time exploring objects during the trial session (E, G) and the test session (F, H) in a novel object recognition task at 2 and 4 weeks (*n* = 7 per group). The data shown in each column represent the mean ± SEM. *p < 0.05, **p < 0.01 with a paired Student's *t*‐test (familiar vs. novel for each group).

We investigated whether decoy peptides alleviate memory loss in αSyn PFF‐injected mice. Peptides were synthesized based on the αSyn C‐terminal and contained 10 residues. Peptide I was identical to residues 131–140 of αSyn, while Peptide II substituted phenylalanine (F) for tyrosine (Y; Tyr133, Tyr136) (Figure 2A). Mice were treated with Peptide I or II one week after receiving the injection of αSyn PFFs, and the cognitive‐related behavioral tests were performed at 2 and 4 weeks, followed by immunohistochemical analysis (Figure 2B). In the Y‐maze test, we observed significantly reduced spontaneous alternation behavior in αSyn PFF‐injected mice relative to normal mice (2 weeks: *p* = 0.0003 vs. control mice). This memory impairment was significantly restored after 1 week of Peptide I treatment (50 μg/kg, *p* = 0.027; 150 μg/kg, *p* = 0.011 vs. vehicle‐treated αSyn PFF mice; Figure 3A) but not Peptide II (150 μg/kg, *p* = 0.081 vs. vehicle‐treated αSyn PFF mice). Following 3 weeks of continuous treatment, both Peptide I‐ and Peptide II‐treated mice exhibited spontaneous alternation behaviors identical to those of normal mice (50 μg/kg and 150 μg/kg, *p* < 0.001 vs. vehicle‐treated αSyn PFF mice; Figure 3C). Additionally, no consistent trend was observed in the number of total arm entries in each group, and although αSyn PFF‐injected mice were less active at 2 weeks (*p* = 0.024 vs. control mice; Figure 3B), they maintained the same state as normal mice at 4 weeks (Figure 3D).

Furthermore, in the novel object recognition test, no difference was observed in the percentage of time spent exploring the two identical objects in each group in the trial session (Figure 3E–G); however, vehicle‐treated αSyn PFF mice failed to discriminate between the familiar and novel objects during the test session (2 weeks: *p* = 0.196, *t* = 1.453, df = 6, Figure 3F; 4 weeks: *p* = 0.441, *t* = 0.826, df = 6, Figure 3H). This aberrant state was ameliorated by both short‐ and long‐term treatment with Peptide I (2 weeks: 50 μg/kg, *p* = 0.044, *t* = −2.534, df = 6; 150 μg/kg, *p* = 0.005, *t* = −4.242, df = 6; 4 weeks: 50 μg/kg, *p* < 0.001, *t* = −6.107, df = 6; 150 μg/kg, *p* = 0.002, *t* = −4.982, df = 6) or Peptide II (2 weeks: 50 μg/kg, *p* = 0.285, *t* = −1.174, df = 6; 150 μg/kg, *p* < 0.001, *t* = −8.430, df = 6; 4 weeks: 50 μg/kg, *p* = 0.015, *t* = −3.384, df = 6; 150 μg/kg, *p* = 0.007, *t* = −3.959, df = 6). Taken together, these observations revealed that αSyn peptide treatment effectively prevented αSyn PFF‐induced memory impairment in mice.

### 3.3 αSyn peptides suppressed phosphorylation of αSyn in OB

**FIGURE 4:**
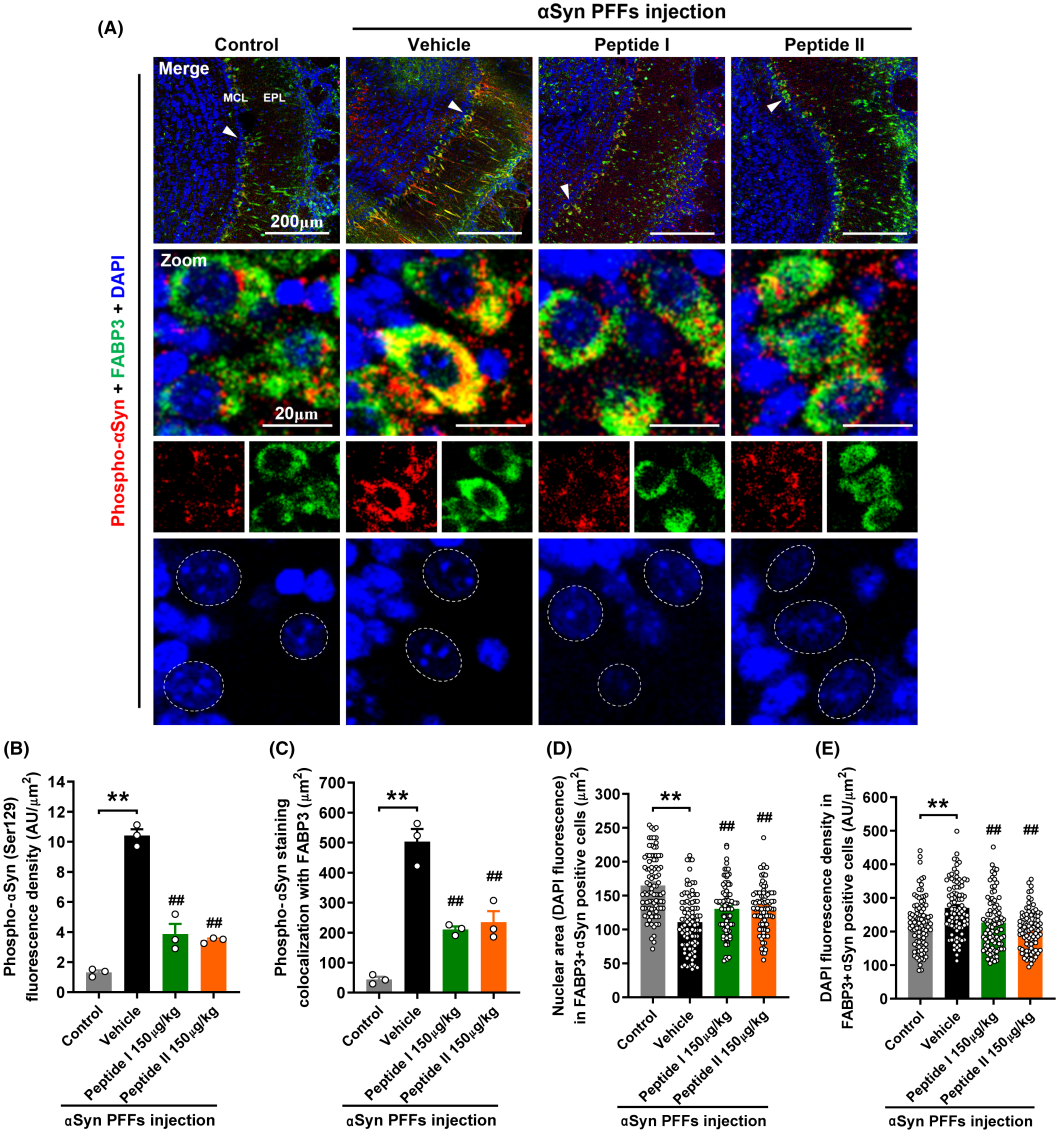
Effects of peptide treatments on αSyn PFF‐induced phosphorylation of αSyn in the olfactory bulb. (A) Representative images of fluorescence immunostaining for phosphorylated αSyn (Ser129) (red), FABP3 (green), and DAPI (nuclei, blue). Quantitative analysis of phosphorylated αSyn (Ser129) immunofluorescence intensity (B) and the areas of phosphorylated αSyn fluorescence colocalized with FABP3 (C) (*n* = 3 mice per group). (D, E) Quantification of DAPI immunofluorescence area (D) and intensity (E) in cells colocalized with phospho‐αSyn and FABP3 (*n* = 90 cells from 3 mice per group). The data shown in each column represent the mean ± SEM. **p < 0.01 vs. control group. ##*p* < 0.01 vs. vehicle‐treated αSyn PFF injection group. Low‐magnification scale bars, 200 μm; high‐magnification scale bars, 20 μm. EPL, external plexiform layer; MCL, mitral cell layer.

αSyn undergoes various post‐translational modifications, especially phosphorylation at serine 129 (Ser129), which affects its aggregation rate and toxicity, as well as pathological spread. In Lewy bodies, more than 90% of αSyn is phosphorylated at Ser129, whereas only 4% is modified in the normal brain.^31^, ^32^ In the OB of αSyn PFF mice, we also observed an increase in phosphorylated αSyn (Ser129), particularly in FABP3‐positive neurons in the mitral cell layer (MCL) (Figure 4A). Treatment with either Peptide I or Peptide II significantly decreased αSyn phosphorylation and even disrupted the binding of αSyn to FABP3 (Figure 4B,C). These in vivo results were consistent with our previous in vitro results, further verifying the inhibitory effect of these peptides on αSyn aggregation. Then, the toxicity of αSyn PFFs to neurons was investigated further by analyzing the morphological changes in the nuclei after DAPI staining. In comparison with neuronal nuclei in control mice, chromatin condensation was clearly observed in αSyn‐accumulated neuronal cells (phospho‐αSyn+FABP3‐positive cells) in αSyn PFFs‐injected mice (Figure 4A), which is one of the morphological characteristics of apoptosis, including a decrease in fluorescent area (Figure 4D) and an increase in intensity (Figure 4E), indicating the strong neurotoxicity of αSyn PFFs on neurons. However, Peptide I or Peptide II treatment effectively reduced the occurrence of chromatin condensation, suggesting that peptides protected neurons against αSyn PFFs.

**FIGURE 5:**
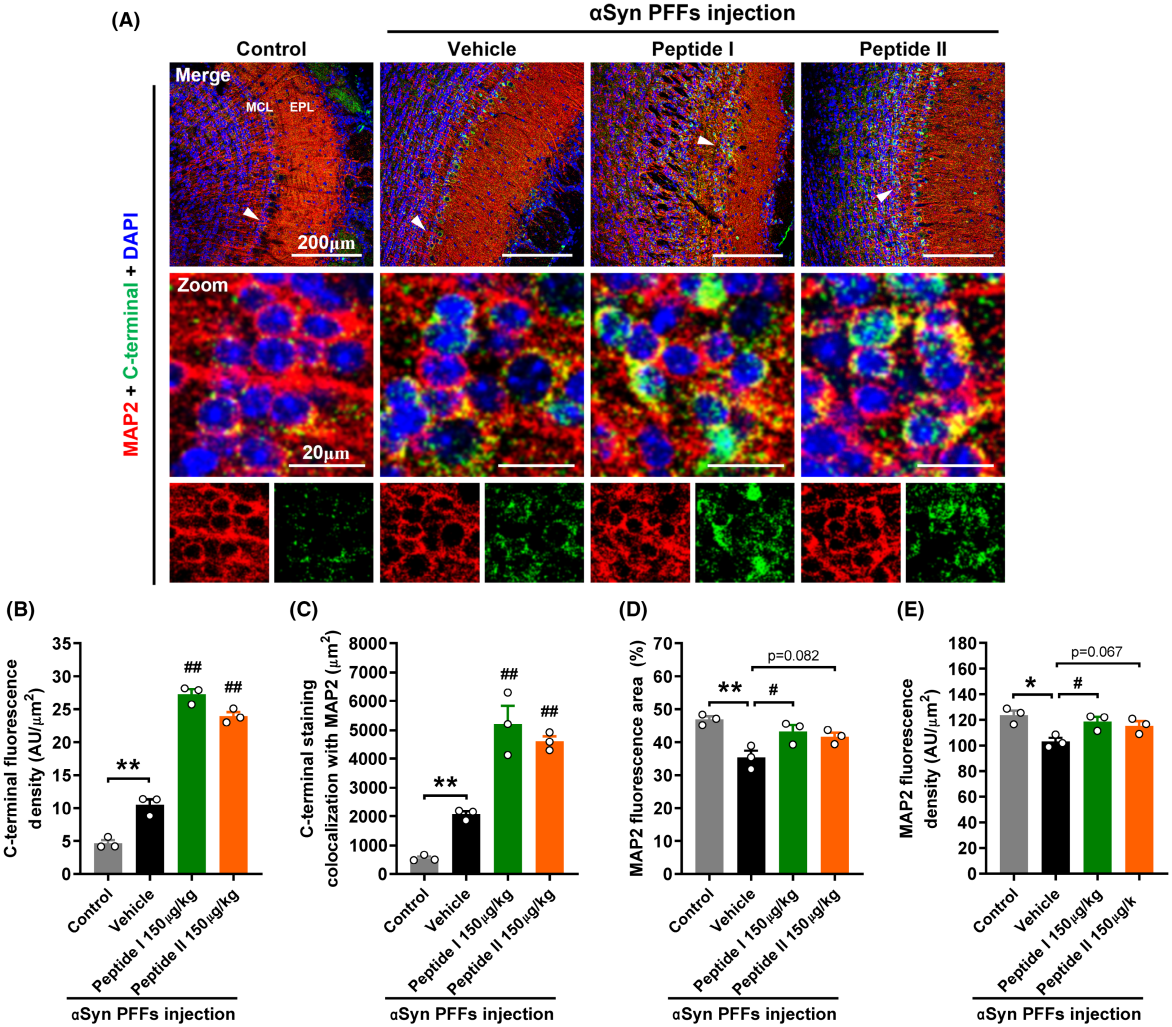
Distribution of peptide in mouse olfactory bulb. (A) Representative images of fluorescence immunostaining of MAP2 (neural marker, red), αSyn C‐terminal antibody (green), and DAPI (nuclei, blue). (B‐C) Quantitative analysis of αSyn C‐terminal immunofluorescence intensity (B) and the areas of αSyn C‐terminal fluorescence colocalized with MAP2 (C). (D, E) Quantification of MAP2 immunofluorescence area (D) and intensity (E). The data shown in each column represent the mean ± SEM. **p < 0.01 vs. control group. ##*p* < 0.01 vs. vehicle‐treated αSyn PFF injection group (*n* = 3 mice per group). Low‐magnification scale bars, 200 μm; high‐magnification scale bars, 20 μm. EPL, external plexiform layer; MCL, mitral cell layer.

Furthermore, to test whether the intranasal administration of peptides was successful, we created an antibody that specifically recognizes the C‐terminal tail of αSyn. According to our quantitative investigation, both Peptide I and Peptide II reached the OB through the nasal cavity (Figure 5A) and maintained a high concentration level (Figure 5B), the majority of which was accumulated in MAP2‐positive neuronal cells (Figure 5C) in the MCL. Meanwhile, immunofluorescence results showed that MAP2 levels in αSyn PFFs mice were reduced (Figure 5D,E), indicating that neurons were destroyed. Together, these findings revealed that the peptides were successfully delivered to the location of αSyn phosphorylation via nasal delivery and exerted effective intervention.

### 3.4 αSyn Peptide I inhibited the binding of αSyn to FABP3

**FIGURE 6:**
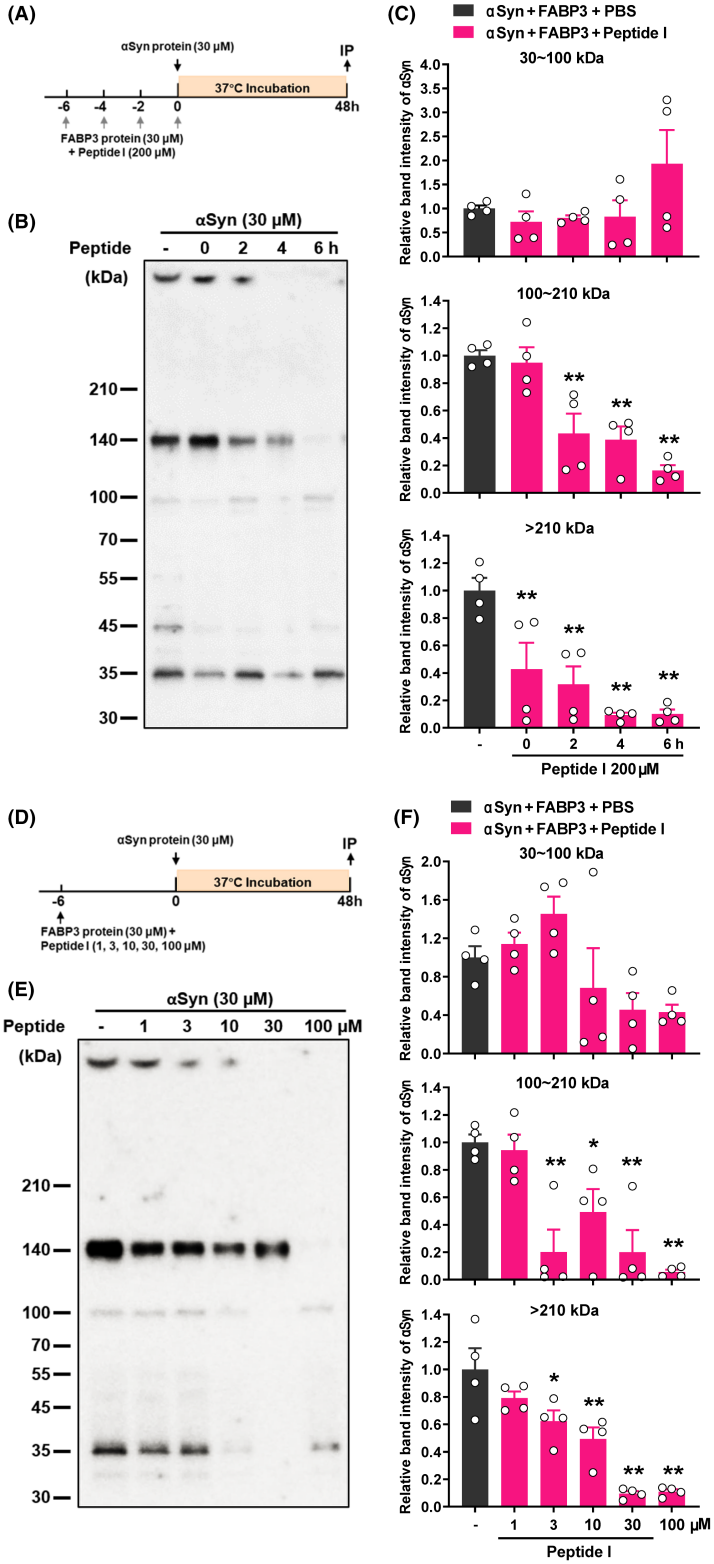
Effects of Peptide I on the binding of αSyn monomer to FABP3 protein. (A) Experimental schedules for time‐dependent effects of the peptide. FABP3 (30 μM) was pre‐incubated with Peptide I (200 μM) for 0, 2, 4, or 6 h at 37°C, and then, αSyn protein (30 μM) was added to continue incubation for 48 h. (B) Representative images of western blots probed with anti‐αSyn antibody. After 48 h of incubation, immunoprecipitation with FABP3 antibody and immunoblotting with αSyn antibody were performed. (C) Quantitative analysis of αSyn‐specific band intensities of different molecular weights, which indicate protein complexes of dimer/trimers/tetramer (30–100 kDa), oligomers (100–210 kDa), and aggregates (>210 kDa). (D) Experimental schedules for concentration‐dependent effects of Peptide I. FABP3 (30 μM) was pre‐incubated with various concentrations of Peptide I (1, 3, 10, 30, and 100 μM) for 6 h at 37°C, and then, αSyn protein (30 μM) was added. (E) Representative images of western blots probed with anti‐αSyn antibody. (F) Quantitative analyses of αSyn‐specific band intensities of different molecular weights. The data shown in each column represent the mean ± SEM. *p < 0.05, **p < 0.01 vs. αSyn + FABP3 + vehicle (*n* = 4 per group).

We next examined whether the presence of these peptides could prevent the formation and expansion of the αSyn‐FABP3 complexes. After incubating αSyn recombinant protein (30 μM) with FABP3 recombinant protein (30 μM) for 48 h, a large number of αSyn‐FABP3 dimer/trimers/tetramer (30–100 kDa), oligomers (100–210 kDa), and aggregates (>210 kDa) were formed, with oligomers accounting for the vast majority (Figure 6A,B). However, if the FABP3 protein and Peptide I (200 μM) were pre‐incubated for 2–6 h, the number of oligomers was significantly reduced, and aggregate growth was also disrupted (Figure 6C). Even with simultaneous incubation, oversized aggregates were greatly decreased. In addition, because the effect of incubating the peptide with FABP3 6 h earlier was the strongest, we evaluated the effects of different peptide concentrations at this time (Figure 6D). The higher concentration of Peptide I showed a stronger blocking effect on the formation of oligomers and aggregates, but no greater effect was observed at 100 μM than at 30 μM. We speculate that when the concentration of Peptide I to FABP3 and αSyn is at a 1:1:1 molar ratio, its inhibitory effect will tend to saturate. These results suggested that the intervention of αSyn decoy peptide substantially inhibited the formation and growth of αSyn‐FABP3 oligomers.

## 4 DISCUSSION

In this study, decoy peptides derived from residues 131–140 of the C‐terminal end of αSyn were found to be resistant to oligomerization and neurotoxicity of αSyn. We observed that peptide treatment ameliorated αSyn PFF toxicity‐induced memory impairment in mice, as well as phosphorylation of αSyn in the OB. We then confirmed the considerable inhibitory effect of αSyn peptides on the formation of αSyn‐FABP3 oligomers. Based on these findings, we propose a novel therapeutic strategy that employs residues from the C‐terminal of αSyn to block its own oligomerization.

The double‐hit hypothesis of PD suggests that αSyn pathology in PD patients first develops in the OB and enteric nervous system.^33^, ^34^ The OB, as the first stage of processing in the olfactory system and in close contact with the outside world, serves as an entry point for pathogens or environmental damage likely to trigger pathological changes in αSyn, which are then propagated throughout the brain via olfactory neural pathways.^35^, ^36^ We injected αSyn PFFs into the OB and observed significant memory deficits in mice starting at 2 weeks after injection, but no motor impairment was observed until 6 weeks later. These behavioral changes were consistent with previous reports^37^, ^38^, ^39^ that the early toxic consequences of αSyn PFFs in the OB mainly involve olfactory deficits, anxiety, and memory impairment, without motor dysfunction, depressive‐like behavior, or circadian rhythm disturbances. αSyn PFFs in the OB did not induce motor dysfunction under a short time due to insufficient diffusion of αSyn, because the main motor symptoms of PD are caused by the death of dopaminergic neurons in the substantia nigra pars compacta,^40^ and the progression of αSyn pathology from the OB to this area takes at least 9 months.^23^, ^41^ Obviously, a 6‐week intervention is not long enough to cause significant changes. At 3 months after injection, Ser129 phosphorylated αSyn is restricted to the olfactory structures and the anatomically connected brain regions, including the piriform cortex, entorhinal cortex, anterior olfactory nuclear, and hippocampus.^23^, ^41^, ^42^ However, αSyn PFF mice displayed transient abnormal motor function 2 weeks after injection (Figure 1E), accompanied by a reduction in locomotor activity (lower total arm entries; Figure 3B). These phenomena have not been fully explained, perhaps this is a normal phenomenon when the acute effects related to the surgical procedure dissipate, and if so, it may explain why these prominent behavioral abnormalities disappeared by weeks 4 and 6. There is also a pathological possibility that αSyn PFFs in the OB induced memory loss and anxiety‐like behaviors in mice,^39^ causing rustiness or fear of forced movements on the rotarod.

In vitro and in vivo experiments demonstrate that FABP3 not only binds to αSyn to keep it in the most neurotoxic oligomeric state^19^ but also contributes to the dissemination of αSyn in the brain.^16^, ^17^ Therefore, hindering the interaction between αSyn and FABP3 is critical to avoid dangerous events from αSyn solution. We previously developed a series of inhibitors of FABP3 and demonstrated that they are significantly resistant to the toxic effects of αSyn.^14^, ^15^, ^43^ As research has progressed, we found that FABP3 was only interested in the C‐terminal tail of αSyn and had no affinity for the truncated version of the C‐terminal tail (αSynΔ130–140) or residues in the N‐terminal (αSynP_2–10_) and NAC regions (αSynP_73–96_). Therefore, we chemically synthesized a decoy peptide (αSynP130–140) from the tail residues and discovered that it competitively hindered the binding of αSyn to FABP3 and reduced the cytotoxicity in Neuro‐2A cells.^19^ In this study, the decoy peptides derived from 10 residues in the C‐terminal tail of αSyn prevented the binding of αSyn to FABP3 in the OB and effectively ameliorated the memory deficit in the αSyn PFF mouse model of PD, confirming that αSyn peptides have a great affinity for FABP3 and prevent αSyn toxicity in vivo. In addition, the importance of the aromatic residue of C‐terminal tyrosine in fibril formation^44^, ^45^ and the replacement of Tyr133 and Tyr136 in αSyn130–140 (EEGYQDYEPEA) with Phe (Y133F/Y136F) results in a three‐fold enhanced affinity for FABP3 (*K*_d_ value changed from 6.1 nM to 2.1 nM).^19^ However, although Peptide II had a significant effect on the improvement of memory impairment and the attenuation of αSyn phosphorylation in mice, the effect was not significantly different from that of Peptide I. One potential reason is that the concentration of Peptides I and II in the OB was substantially higher than that of αSyn PFFs due to their continuous administration, which was insufficient to reflect the difference in their effects. Furthermore, we will further investigate the impact of these peptides on the motor abnormalities, cognitive impairment, and intracerebral transmission of αSyn caused by αSyn PFF injection in the striatum or substantia nigra.

In PD patients with Lewy bodies, more than 90% of abnormally aggregated αSyn is phosphorylated at Ser129,^46^, ^47^, ^48^ which is thought to have pathological significance. Phosphorylation here is also widely recognized as a major source of aggregation and neurotoxicity.^49^, ^50^, ^51^ αSyn PFFs activated the phosphorylation of αSyn Ser129 in FABP3‐positive neuronal cells in the MCL area of the OB, accompanied by a significant number of αSyn‐FABP3 complexes. With the intervention of these peptides, the occurrence and development of these pathogenic alterations were strongly disrupted. Considering that depletion of FABP3 avoids phosphorylation of αSyn,^16^ we hypothesized that peptides blocked αSyn phosphorylation at Ser129 by first depleting large amounts of FABP3. DAPI staining also found that the chromatin was obviously condensed in FABP3‐positive neurons with a large amount of phosphorylated αSyn, indicating that neurons were undergoing apoptosis; however, this morphological feature of apoptosis was not significant in peptide‐treated mice, suggesting that peptide can protect neurons against αSyn‐induced neurotoxicity. Meanwhile, the quantitative findings of the neuronal dendrite marker MAP2 also supported this conclusion. Furthermore, using an anti‐αSyn C‐terminus‐specific antibody to detect the localization of exogenous peptides, quantitative analysis revealed that peptides were also mostly deposited in the neuronal cells in the MCL, with an excellent ability to target cells damaged by αSyn toxicity. Meanwhile, we observed immunofluorescence in the OB of control and vehicle‐treated αSyn PFF mice (Figure 5A), although they were not treated with peptides. This is because the antibody recognizes the C‐terminus of αSyn but does not specifically target peptides. In fact, αSyn is widely expressed in the nervous system, accounting for 1% of the total cytosolic proteins.^52^, ^53^ The immunofluorescence intensity in the OB of vehicle‐treated αSyn PFF mice was higher than that of control mice, due to the increase in αSyn aggregation/expression stimulated by PFFs.^54^ Meanwhile, the fluorescence intensity in mice‐treated long term with peptides was significantly higher than that in vehicle‐treated αSyn PFF mice, at least 60% of which were likely to be exogenous Peptide I or II (Figure 5B). However, our results are not sufficient evidence that the peptides can cross the blood–brain barrier. Future experiments with fluorescently labeled peptides will be valuable in confirming the results presented here. Furthermore, FABP3 is essential for the uptake and propagation of αSyn for its accumulation in dopaminergic neurons, which rely on caveola formed by dopamine D_2L_ receptors, FABP3, and caveolin‐1 to uptake αSyn monomers and fibrils.^55^, ^56^ Additionally, FABP3 can bind to the dopamine D_2L_ receptor and regulate its function.^57^, ^58^ Although how peptide binding impacts FABP3 function is unclear, the peptide may strike FABP3 in a functionally inhibitory manner, disrupting the construction of caveola and preventing the neurons in the OB from reacting to the uptake and accumulation of αSyn PFFs. We will continue to further investigate whether peptides affect the uptake of αSyn by dopaminergic neurons.

All these findings suggested that peptides could reduce the phosphorylation and neurotoxicity of αSyn, providing a great possibility for clinical application in vivo. However, in addition to effectiveness, another important factor limiting the clinical applicability is toxic side effects. The C‐terminus of αSyn (residues 96–140) is a flexible and disordered structure with high acidity. The charged status and structure of this region allow αSyn to interact with metals, positively charged proteins and membrane binding,^59^, ^60^ such as Fe^3+^,^61^ Ca^2+^,^62^ tau protein,^63^ calmodulin,^64^ synaptic vesicles,^62^ affecting Ca^2+^ homeostasis or forming toxic aggregates. Peptides derived from the C‐terminus were also acidic (residues 131–140, **E**GYQ**D**Y**E**P**E**A, include 4 acidic amino acids). Therefore, peptides may have impacts on the homeostasis of calcium ions and synaptic vesicles, as well as other cationic ligands. Fortunately, Oikawa, T et al discovered that αSyn monomer did not interact with tau,^60^ suggesting that peptides may not bind to tau or interfere with microtubule formation. Furthermore, peptides appear to be safe at least at short and intermediate term, because no adverse reactions were observed in peptide‐treated mice over the course of our study, although our observations were confined to diet, hair, vitality, memory, and motor behavior. Further experiments will be performed to evaluate its safety and toxicity. In addition, the pharmacokinetics of peptide in vivo also need to be further clarified. Although shorter peptides are more stable without affecting efficacy, several proteases can cut and degrade peptides, including cathepsin D (CtsD, cleavage site: G132/Y133) in lysosomes^65^ and extracellular matrix metallopeptidase 1 (MMP1, Y133/Q134).^47^

αSyn‐FABP3 oligomers are an important pathogenic manifestation of αSyn neurotoxicity. Following in vitro co‐incubation of these two recombinant proteins, a significant number of oligomers were formed (molecular weight around 140 kDa, and likely composition (αSyn‐FABP3)_5_), as well as less αSyn‐FABP3 dimers and larger aggregates. However, Peptide I from the C‐terminus competitively bound to FABP3, hindering the binding of αSyn‐FABP3 and greatly reducing the formation of these complexes. This novel finding suggests that αSyn fragments can hinder their own aggregation and possess good therapeutic effects in vivo. It also points to a new direction for the development of therapeutic drugs for α‐synucleinopathies. In addition, the interaction of this peptide with FABP3 involves a specific phenylalanine residue (F16) in FABP3, because F16 mutation renders FABP3 incapable of binding to PUFAs or αSyn.^13^, ^19^ Moreover, the mutation of Y133 or Y136 on the peptide affects its affinity for FABP3.^19^ We intend to further confirm the structure and interaction of the αSyn‐FABP3 complex using X‐ray crystallography or NMR in the future.

**FIGURE 7:**
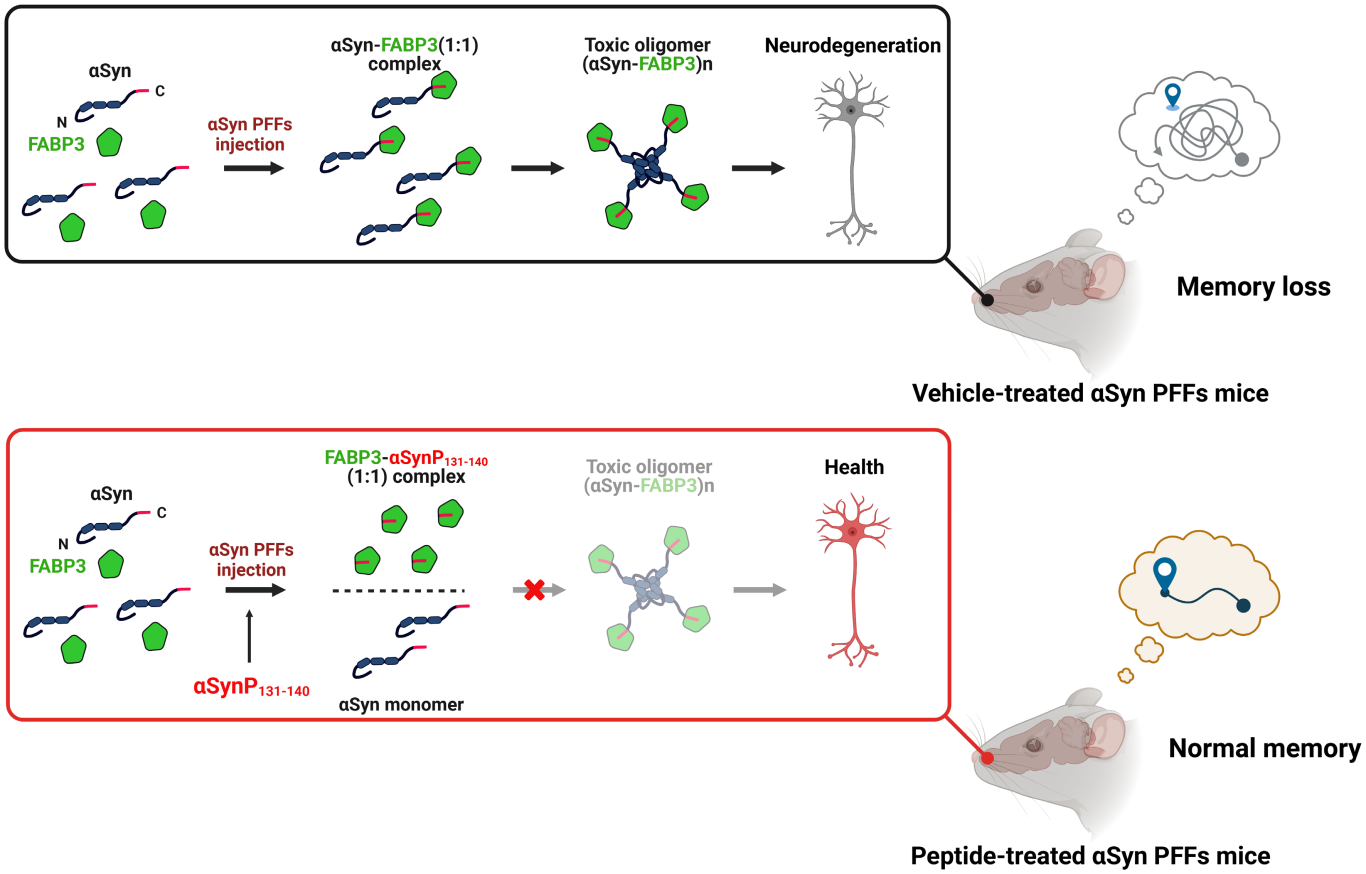
Schematic diagram of toxicαSyn‐FABP3 oligomer formation in the olfactory bulb (OB) and memory loss in αSyn PFF mice. (Top) Following injection of αSyn PFFs into the mouse OB, αSyn phosphorylates and complexes with FABP3 to form toxic oligomers, causing neuronal degeneration and memory loss in mice. (Bottom) Peptides (αSynP131–140) competitively bind to FABP3, limiting the development of toxic αSyn‐FABP3 oligomers, protecting neurons, and avoiding memory impairment. Figure was created with BioRender.com.

In this study, we confirmed that injecting αSyn PFFs into the OB impairs memory but not motor dysfunction in mice and that αSyn decoy peptides significantly restore memory loss in the αSyn PFF mouse model by binding to FABP3 to block the phosphorylation/aggregation of endogenous αSyn and avoid the formation of toxic αSyn‐FABP3 oligomers (Figure 7). Our findings provide novel insights into the therapeutic potential of αSyn peptides for α‐synucleinopathies.

## AUTHOR CONTRIBUTIONS

Qingyun Guo: investigation and original manuscript writing. Ichiro Kawahata and Yasushi Yabuki: investigation and methodology. Wenbin Jia, Haoyang Wang, An Cheng, and Norifumi Shioda: investigation. Kohji Fukunaga: supervision, review/editing, project administration, and funding. All authors reviewed the final manuscript and approved it for publication.

## CONFLICT OF INTEREST STATEMENT

I.K. received a research grant from Takeda Science Foundation, and Smoking Research Foundation. The other authors (Q.G., W.J., H.W., A.C., Y.Y., N.S., K.F.) declare no conflicts of interest in the present study.

## Supporting information


AppendixS1

## Data Availability

The data that support the findings of this study are available from the corresponding author upon reasonable request.
