# Supplementary material for: α‐Synuclein decoy peptide protects mice against α‐synuclein‐induced memory loss
Source: CNS Neurosci Ther. 2023 Feb 14;29(6):1547–60. doi: 10.1111/cns.14120 (PMC10173724; doi:10.1111/cns.14120)

The specificity of homemade αSyn C-terminal antibody

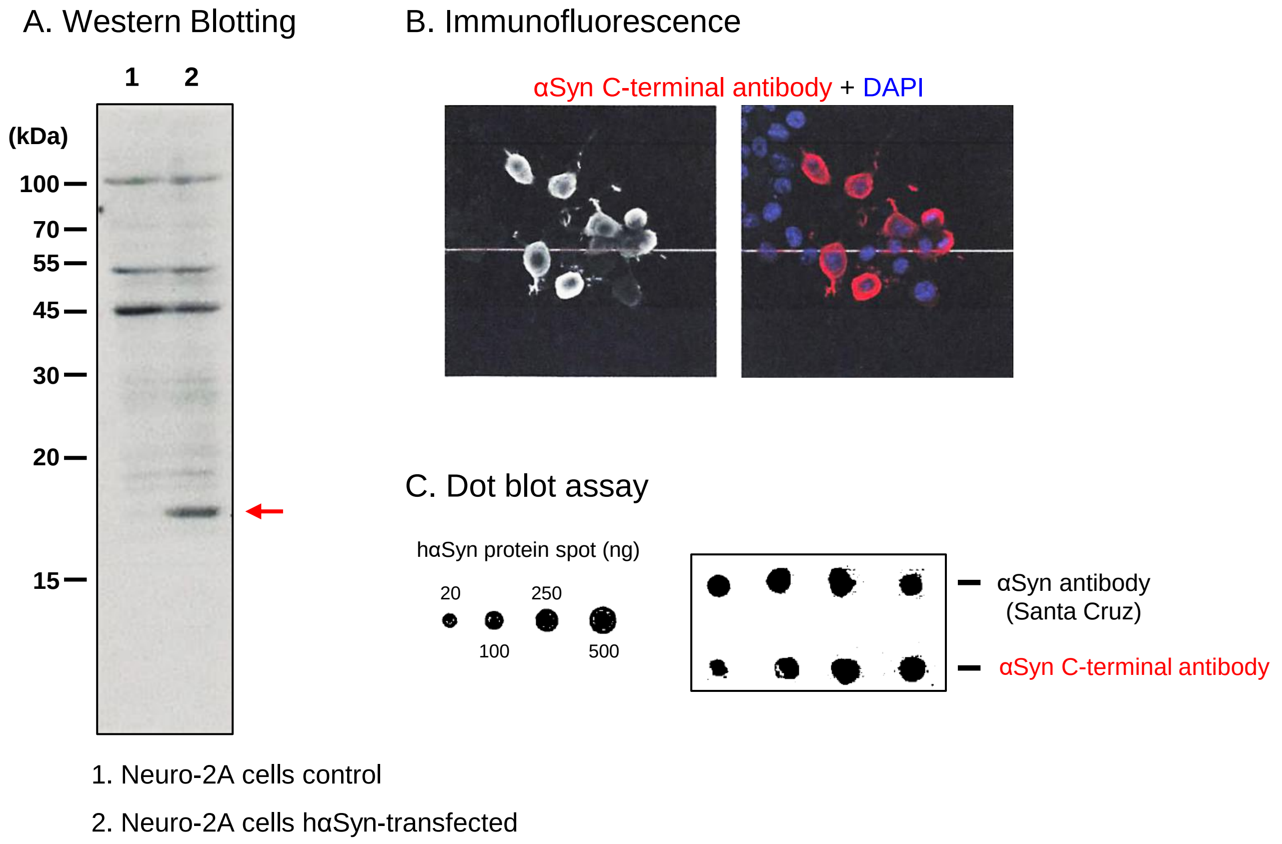

Specific detection of αSyn C-terminal antibody. (**A**) Representative images of western blots shows lysates of Neuro-2A mouse neuroblastoma cell line (1) and human αSyn-transfected Neuro-2A cells (2). PVDF membrane was probed with mouse anti-human αSyn C-terminal antibody followed by HRP-conjugated anti-mouse IgG secondary antibody. A specific band was detected for αSyn at approximately 18 kDa (as indicated). **(B)** Representative image of fluorescence immunostaining for αSyn C-terminal antibody (red), and DAPI (nuclei, blue) in human αSyn-transfected Neuro-2A cells. **(C)** Dot blot assay was used to evaluate the level of recombinant human αSyn protein incubated with αSyn C-terminal antibody or commercially available αSyn antibody (Santa Cruz, Cat# sc-12767, RRID:AB_628318, CA, United States). Left Panel: human αSyn protein standards, amount of protein (ng) is indicated above each spot. Right Panel: representative dot blot image.

Original western blot images

Full unedited blot for Fig.5B

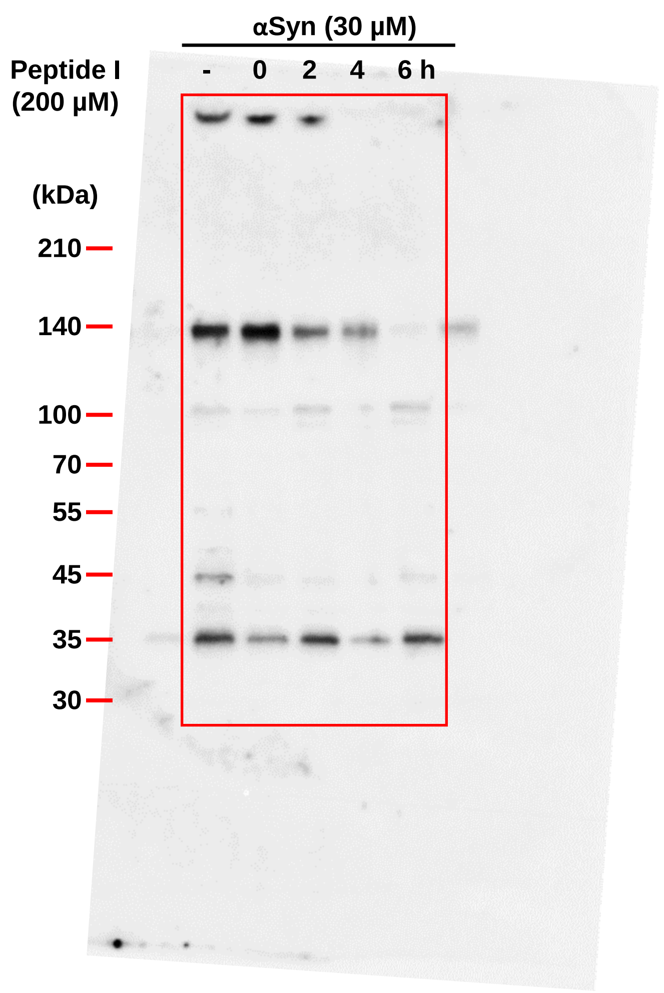

Full unedited blot for Fig.5E

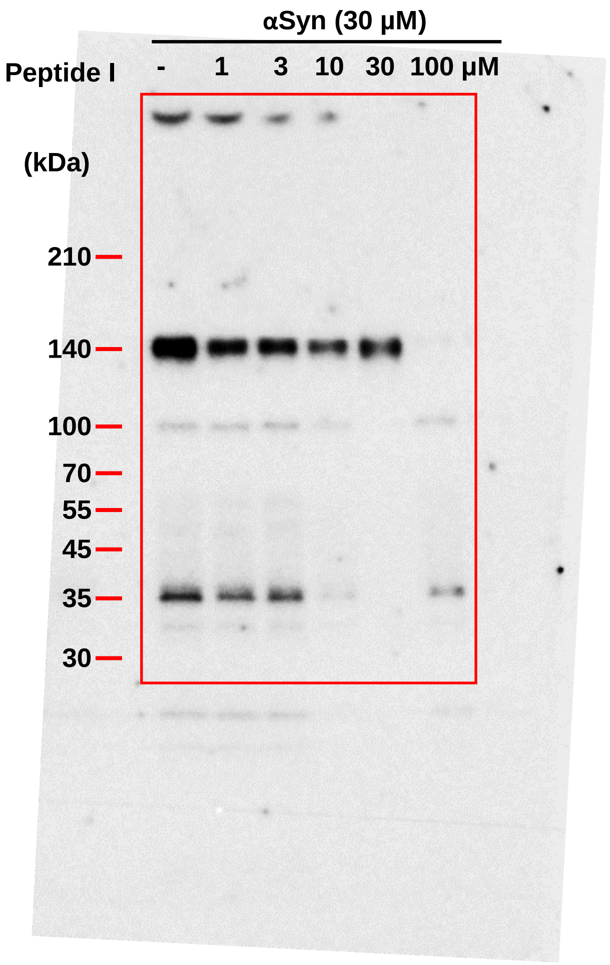

Supplement: Supplementary file 1 — AppendixS1 [file CNS-29-1547-s001.pdf]
